# Optimal Systemic Treatment of Advanced Bladder Cancer—A Moving Target

**DOI:** 10.3390/cancers12123607

**Published:** 2020-12-02

**Authors:** Helle Pappot, Anders Ullén

**Affiliations:** 1 Department of Oncology, Team Urooncology, Rigshospitalet, University Hospital of Copenhagen, Blegdamsvej 9, 2100 Copenhagen, Denmark; 2 Department of Pelvic Cancer, Theme Cancer, Karolinska Sjukhuset, Karolinska University Hospital, 17176 Stockholm, Sweden; 3 Department of Oncology-Pathology, Karolinska Institute, 17176 Stockholm, Sweden

Bladder cancer (BC) is diagnosed in more than 500,000 cases annually worldwide, and it represents the sixth most common malignancy in the Western world. 

For many years the treatment for advanced bladder cancer has been limited, mostly restricted to one or two lines of systematic treatment, but new technologies and general achievements within oncology are opening new possibilities and also the chance for prolonged life expectancy for bladder cancer patients.

The introduction of immunotherapy and targeted therapies has initially been restricted to large disease entities like breast, gastrointestinal and lung cancer, and some specific diseases like malignant melanoma and renal cell carcinoma, but recently the benefits of such therapies have also been confirmed for patients with advanced bladder cancer.

Until the introduction of cisplatinum in 1978, no effective treatment resulting in long-term survival was available to control advanced bladder cancer, and even with this active drug available, it took more than two decades to develop effective combination chemotherapy and decide the scheduling thereof [1].

Despite the introduction of platinum in the handling of advanced bladder cancer and attempts to optimize combination regimens, survival rates have remained low with a five-year survival limited to 15% [2,3]. For platinum-progressive patients, taxanes have commonly been used, but these compounds have never been approved for this purpose and treatment efficacy has not been convincing. In 2009, the microtubule inhibitor Vinflunine was approved and introduced as the first specific second-line chemotherapy, albeit with limited survival benefit [4]. 

The high frequency of somatic mutations associated with bladder cancer, similar to melanoma and lung cancer, led to high expectations of a benefit from immunotherapy with check-point inhibitors (ICIs), which had shown activity in other diagnoses. In 2016, this new treatment modality was introduced also in advanced bladder cancer [5]. Improvement of survival was awaited through this shift in treatment, but probably due to the heterogenetic nature of BC, lower median survival outcomes and response-rates have been demonstrated compared to other diseases such as malignant melanoma and renal cell carcinoma [5,6,7,8,9,10,11,12,13]. Nevertheless, ICIs have been a breakthrough and the first real paradigm shift in advanced bladder cancer treatment in nearly a half century. One of the major challenges in BC treatment is that only approximately 20–25% of advanced BC patients seem to respond to ICIs and no gold standard for selecting those patients most likely to benefit from ICIs treatment exists. This calls for a better understanding of bladder cancer biology, biomarker discovery and development of selection methodologies which are likely to have a clinical impact on bladder cancer treatment and outcomes. 

At present, optimal systemic treatment of advanced bladder cancer is a moving target. The very recent advances introduce: alongside immunotherapy using Avelumab in a switch maintenance approach after first-line chemotherapy, targeted therapies in the second and third lines, which gives further hope for improved overall survival and personalized treatment management of bladder cancer patients in the future [14]. Specifically, the pan-FGFR tyrosine-kinase inhibitor Erdafitinib and the first-in-class anti-Nectin-4 antibody drug conjugate Enfortumab-Vedotin was recently approved by the FDA in the US as the first two systemic targeted therapies for this disease [15,16] and we expect more to come; currently more than 200 clinical trials are ongoing in advanced bladder cancer [17]. The optimal sequence and treatment combinations of these new compounds with different mechanisms of action are yet to be defined and are a matter of priority for the research community. 

Moreover, we believe that the cancer trajectory for a patient with advanced bladder cancer receiving systemic treatment may be influenced by multiple factors, with the outcome dependent on both new biological knowledge as well as optimal supportive care. Therefore, improved symptom management and attempts to better monitor and optimize quality of life in this, often vulnerable, patient population must not be forgotten.
